# Predictive value of electrocardiogram Morphology-Voltage-P wave duration score for new episodes of atrial fibrillation in patients with acute myocardial infarction: a retrospective study

**DOI:** 10.3389/fcvm.2026.1714839

**Published:** 2026-03-04

**Authors:** Yangxue Li, Jiangen Liu, Yang Lu, Bin Liu

**Affiliations:** Department of Cardiology, The Second Hospital of Jilin University, Changchun, China

**Keywords:** acute myocardial infarction, long-term prognosis, MVP ECG score, new-onset atrial fibrillation, risk prediction, riskstratification

## Abstract

**Background:**

The prevalence of atrial fibrillation (AF) is approximately 1.5%–2% of the general population. The Morphology-Voltage-P Wave Duration (MVP) score, a novel electrocardiographic tool based on *P*-wave characteristics, has shown promise in predicting AF risk. New-onset atrial fibrillation (NOAF) following acute myocardial infarction (AMI) is associated with poor clinical outcomes.

**Methods:**

This retrospective study included 334 patients treated for AMI from January 2018 to April 2021. Patients were categorized into low-risk (202 cases), medium-risk (98 cases), and high-risk (34 cases) groups based on MVP ECG scores. NOAF incidence, stratified as early-onset (≤48 h) and late-onset (>48 h), was tracked over a 12-month follow-up period. Statistical analyses included group comparisons, Cox regression for multivariate adjustment, ROC analysis to evaluate and compare the predictive performance of the MVP score against both the CHA₂DS₂-VASc and the AF-specific C2HEST scores, and decision curve analysis (DCA) to assess clinical utility.

**Results:**

The MVP ECG risk score effectively predicted long-term AF incidence, showing a graded increase in risk across categories: 11.9% in low-risk, 28.6% in medium-risk, and 76.5% in high-risk patients. Incidence increased for both early-onset (4.0%, 10.2%, 29.4%) and late-onset NOAF (7.9%, 18.4%, 47.1%). The MVP score demonstrated superior discriminative ability for total NOAF (AUC = 0.908) compared to the CHA₂DS₂-VASc score (AUC = 0.643) and the C2HEST score (AUC = 0.715), with the highest performance observed for late-onset NOAF (AUC = 0.925). Addition of the MVP score to either clinical score significantly improved reclassification (NRI: 0.28–0.35, IDI: 0.07–0.08). DCA confirmed that using the MVP score provided a greater net clinical benefit than the comparator scores across realistic decision thresholds. Multivariate analysis confirmed the MVP score as an independent predictor of AF, with a stronger association for late-onset events.

**Conclusion:**

The MVP ECG risk score is a simple, non-invasive tool that provides superior prediction of NOAF in AMI patients compared to traditional clinical risk scores, exhibiting particular strength in identifying patients at risk for late-onset AF. It effectively identifies high-risk patients who may benefit from close monitoring and proactive management strategies.

## Visual abstract

**Figure:**
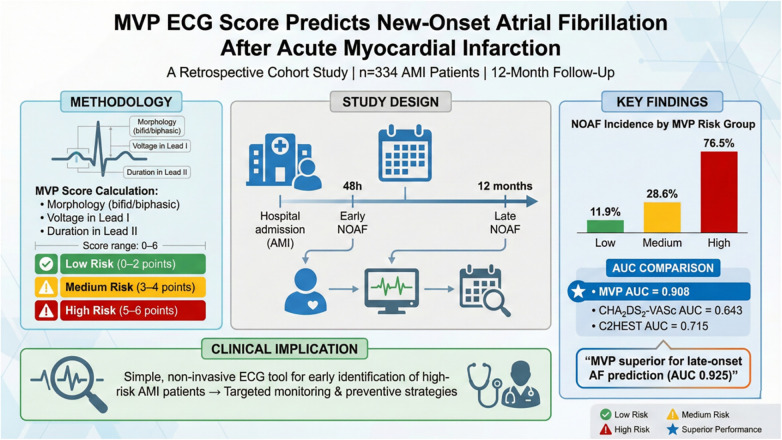
This retrospective study included 334 patients with acute myocardial infarction (AMI) to evaluate the Morphology-Voltage-P wave duration (MVP) ECG score as a predictor for new-onset atrial fibrillation (NOAF). Patients were stratified into low, medium, and high-risk categories based on *P*-wave characteristics measured at admission. Over a 12-month follow-up, the incidence of NOAF showed a significant graded increase: 11.9% in the low-risk group, 28.6% in the medium-risk group, and 76.5% in the high-risk group. The MVP score demonstrated superior discriminative ability (AUC = 0.908) compared to established clinical scores (CHA₂DS₂-VASc: AUC = 0.643; C2HEST: AUC = 0.715), exhibiting exceptional performance for predicting late-onset AF (AUC = 0.925). These findings highlight the MVP score as a simple, non-invasive tool for effective risk stratification and targeted management in post-AMI patients.

## Introduction

Atrial fibrillation (AF), frequently occurring in conjunction with acute myocardial infarction (AMI), is the most prevalent clinical cardiac arrhythmia, with incidence rates between 6% and 21%. Factors predicting arrhythmias related to AMI encompass advanced age, heart failure symptoms, and diminished left ventricular function (1, 2). Substantial evidence suggests that NOAF in patients hospitalized for acute myocardial infarction has a significant adverse impact on both in-hospital and long-term mortality rates (3–6). Atrial fibrillation associated with AMI not only heightens the risk of ischemic stroke while hospitalized but also elevates the risk during follow-up periods (7). Despite these important clinical considerations, the current prediction of NOAF in patients with acute myocardial infarction primarily relies on traditional electrocardiogram analysis or relies on clinical symptoms and cardiac biomarkers, both of which have limitations in terms of sensitivity and specificity (8–12). Furthermore, for patients with AF complicating AMI, specific treatment guidelines that address key issues such as the use of antiarrhythmic drugs, pharmacological control, and thromboembolism prevention are lacking (13, 14). Therefore, there is an urgent need for a sensitive, effective, reliable, and cost-effective detection method (15, 16).

The Morphology-Voltage-P Wave Duration (MVP) score, a detailed scoring system, is based on the morphology, voltage, and *P* wave duration observed in electrocardiograms (17). This relatively new predictive tool, used to forecast atrial fibrillation in patients with coronary artery disease (18), has yet to show its capability in predicting both in-hospital and long-term atrial fibrillation among AMI patients (13, 19). It is worth noting that the MVP score can integrate multiple dimensions of information from the patient's electrocardiogram, and its predictive ability may be more accurate in comparison to single electrocardiogram parameters (20–22). Therefore, MVP score not only has the potential to become a sensitive, effective, reliable, and cost-effective tool for predicting NOAF after AMI; more importantly, its application may facilitate more precise monitoring and management of high-risk patients with AMI, enabling early preventive measures to improve long-term prognosis. This retrospective study was designed to assess the effectiveness of the MVP score in predicting new-onset atrial fibrillation among patients with acute myocardial infarction. By collecting and analyzing retrospectively acquired clinical data from 334 AMI patients, we aimed to validate the predictive value of the MVP score and compare its performance with established risk assessment tools. Furthermore, we investigated the incremental predictive value of combining the MVP score with clinical parameters to develop a more accurate risk stratification model for NOAF, ultimately providing more individualized risk assessment and management strategies for AMI patients.

## Methods

### Study design and populations

This retrospective observational, descriptive longitudinal study seeks to determine the usefulness of the MVP risk score in forecasting the emergence of new-onset AF in AMI patients over the initial 12 months of follow-up. The study was conducted using retrospectively collected data from January 2018 to April 2021 at The Second Hospital of Jilin University, with consecutive enrollment of patients diagnosed with AMI. Inclusion criteria were as follows: Age > 18 years; Clinically and radiographically confirmed AMI; At admission, high-quality ECG and echocardiogram data are available, along with access to follow-up information from the national healthcare system. Exclusion criteria were as follows: History of pre-existing AF (as ascertained by thorough review of all available medical records, including prior ECGs, Holter monitoring reports, and patient self-report); Patients with implanted cardiac devices; Cases with poor ECG quality or unreadable recordings. The MVP ECG risk score was calculated for each patient at the time of admission, and the study population was subsequently grouped according to the resulting risk score.

### Data collection

Data were retrospectively collected from electronic medical records at the time of admission and included demographic information, medical history, laboratory results, and detailed ECG and echocardiogram parameters. Follow-up data were obtained through the hospital's electronic database and included information on the occurrence of new-onset AF and other relevant clinical outcomes.

### ECG and echocardiographic evaluation

All patients underwent standard 12-lead ECG recordings upon admission at a paper speed of 25 mm/s and a calibration of 10 mm/mV. Three independent cardiologists, blinded to all patient clinical data and outcomes, evaluated the ECGs. Prior to analysis, all reviewers participated in a centralized training session to ensure uniform application of the measurement criteria, which were adapted from the established methodology described by Alexander et al. (18). *P*-wave analysis was performed manually on digitally acquired recordings using electronic calipers within the ECG management system. The *P*-wave morphology was assessed in the inferior leads (II, III, and aVF) and categorized as normal (monophasic with a single positive deflection) or abnormal. Abnormal morphology was specifically defined as a bifid (notched) *P* wave, characterized by the presence of two distinct peaks with an interpeak trough > 0.05 mV, or a biphasic pattern (positive-negative deflection). The maximum *P*-wave voltage was measured in lead I from the isoelectric line to the peak of the waveform. *P*-wave duration was measured in lead II from the earliest onset to the latest offset of the *P*-wave relative to the isoelectric line across all leads, and the longest duration was recorded. To ensure accuracy and minimize bias, each ECG tracing was independently analyzed by two cardiologists. Any discrepancies in measurements (defined as a difference in *P*-wave duration > 10 ms, voltage > 0.05 mV, or disagreement in morphology classification) were adjudicated by the third senior cardiologist to reach a consensus. The inter-observer variability was excellent, as assessed by the intraclass correlation coefficient (ICC = 0.92 for duration; ICC = 0.89 for voltage) and Cohen's kappa (*κ* = 0.85 for morphology) in a random sample of 30 ECGs. The MVP risk score was calculated by summing points assigned for three *P*-wave characteristics based on predefined criteria adapted from Alexander et al. (18): (1) Morphology in inferior leads (II, III, aVF): 0 points for normal (monophasic), 2 points for abnormal (bifid or biphasic); (2) Voltage in lead I: 0 points for amplitude < 0.1 mV, 1 point for 0.1 to 0.15 mV, 2 points for > 0.15 mV; (3) Duration in lead II: 0 points for < 120 ms, 1 point for 120 to 200 ms, 2 points for > 200 ms. The total MVP score ranges from 0 to 6. Based on the published parameters integrating these assessments, the MVP score was calculated. Patients were subsequently categorized into three groups: low-risk group (0–2 points), medium-risk group (3–4 points), and high-risk group (5–6 points). The CHA₂DS₂-VASc score was calculated for each patient based on the following criteria: congestive heart failure (1 point), hypertension (1 point), age ≥75 years (2 points), age 65–74 years (1 point), diabetes mellitus (1 point), stroke/transient ischemic attack/thromboembolism history (2 points), vascular disease (1 point; defined as prior myocardial infarction, peripheral artery disease, or aortic plaque), and sex category (i.e., female sex) (1 point). The C2HEST score was also calculated, assigning points as follows: Coronary Artery Disease (1 point), Chronic Obstructive Pulmonary Disease (1 point), Hypertension (1 point), Elderly (age ≥75 years, 2 points), Systolic Heart Failure (2 points), and Thyrotoxicosis (1 point) (23).

### Long-term AF monitoring

The detection of NOAF encompassed an in-hospital phase and a structured 12-month post-discharge follow-up phase. In-hospital detection was based on continuous 72-hour telemetry monitoring initiated immediately upon admission. An AF episode was defined as an irregular rhythm without distinct *P* waves lasting for a minimum of 30 s, confirmed by a cardiologist's review of the telemetry tracings. Following established precedents (24), NOAF was further categorized into early-onset (occurring ≤48 h after admission) and late-onset (>48 h after admission) to differentiate between the distinct pathophysiological mechanisms and prognoses associated with each.

For post-discharge detection during the 12-month follow-up, a multimodal strategy combining scheduled assessments and systematic healthcare records review was implemented. All patients were scheduled for outpatient clinic visits at 1, 3, 6, and 12 months after discharge. Each visit included a standardized interview for arrhythmia-related symptoms such as palpitations or dyspnea, and a resting 12-lead electrocardiogram (ECG). Furthermore, a 24-hour Holter monitor was routinely performed for all patients who remained in follow-up at the 6-month visit. To identify AF episodes occurring between scheduled visits or leading to unscheduled medical care, the national healthcare database was comprehensively interrogated to capture any clinical encounter, including emergency department visits, hospital readmissions, or consultations at other affiliated institutions associated with a documented diagnosis of AF (International Classification of Diseases, 10th Revision code I48). Medical records from these encounters, such as discharge summaries and ECG or Holter reports, were reviewed to confirm the NOAF diagnosis. A diagnosis of long-term NOAF during follow-up was established if AF was confirmed on any scheduled 12-lead ECG, 24-hour Holter report, or in the clinical documentation from a healthcare system encounter identified through the database search. The complete detection protocol is summarized in Table 1. All echocardiographic examinations were performed during the index hospitalization.

**Table 1:** Protocol for detection of New-onset atrial fibrillation (NOAF).

Monitoring phase	Time point/context	Detection method	Application/Scope	NOAF positivity criterion
In-Hospital	From admission	Continuous 72-hour telemetry	All enrolled patients	AF episode ≥ 30 s confirmed on telemetry strip review.
Follow-up (Active)	Scheduled visit: 1, 3, 6, 12 months	Resting 12-lead ECG	All patients attending follow-up	AF confirmed on ECG interpretation report.
Follow-up (Active)	Scheduled visit: 6 months	24-hour Holter monitor	All patients attending the 6-month visit	AF episode documented in the Holter report.
Follow-up (Passive)	Any unscheduled encounter	National healthcare database search (ICD-10: I48)	All patients’ records throughout follow-up	AF diagnosis documented in clinical notes (ED, admission, clinic) from any captured encounter.
Follow-up (Symptom-driven)	Patient-reported symptoms	Additional ECG or event recorder	As clinically indicated during follow-up	AF confirmed on the resulting recording.

AF, atrial fibrillation; ECG, electrocardiogram; ED, emergency department; ICD-10, International Classification of Diseases, 10th Revision.

### Statistical analysis

Data were analyzed to compare the incidence and predictors of NOAF across MVP risk categories. The primary endpoint was the time from admission to the first documented AF episode during the 12-month follow-up. Secondary analyses examined NOAF according to timing of onset: early-onset (≤48 h after admission) and late-onset (>48 h after admission). All analyses were performed using SPSS 25.0 (SPSS Inc., Chicago, IL, USA) and R software (version 4.2.2). The normality of data distribution was assessed using the Kolmogorov–Smirnov test. Categorical data, presented as frequencies and percentages, were compared using Pearson's chi-square test or Fisher's exact test, as appropriate. Non-normally distributed continuous variables, expressed as medians with interquartile ranges (IQR), were compared across MVP risk groups using the Kruskal–Wallis test, with Dunn's test and Bonferroni correction for *post hoc* pairwise comparisons. Incidence rates between groups were also compared using risk ratios with 95% confidence intervals (CI).

Time-to-event analyses were performed using Kaplan–Meier curves with the log-rank test for group comparisons. To identify predictors of NOAF, univariate and multivariate logistic regression analyses were conducted for in-hospital AF. For long-term AF, univariate Cox proportional hazards regression was performed first. Variables with a *P*-value < 0.20 or of established clinical relevance were included in subsequent multivariate Cox regression models. Two primary multivariate Cox models were constructed for total NOAF: Model 1 adjusted for age and sex, and Model 2 additionally adjusted for hypertension, diabetes mellitus, hyperlipidemia, smoking, chronic renal failure, coronary artery disease, CHA₂DS₂-VASc score, left ventricular ejection fraction (LVEF), and left atrial diameter. Separate Cox regression analyses were performed for early-onset and late-onset NOAF using the same adjustment strategy. Results are reported as hazard ratios (HR) with 95% CI. The proportional hazards assumption was verified using Schoenfeld residuals. Further comparisons among MVP risk groups and assessment of potential confounding were performed using multivariate-adjusted and stratified models.

The discriminatory ability of the MVP score, CHA₂DS₂-VASc score, and C2HEST score for predicting NOAF was assessed and compared by calculating the area under the receiver operating characteristic curve (AUC). Performance was evaluated separately for total NOAF, early-onset NOAF, and late-onset NOAF. Pairwise comparisons of AUC values were performed using the DeLong test. The incremental predictive value of the MVP score beyond existing risk scores was evaluated by calculating the net reclassification improvement (NRI) and integrated discrimination improvement (IDI) for both the CHA₂DS₂-VASc and C2HEST scores. The clinical utility of the prediction models was further assessed using decision curve analysis (DCA) to quantify the net benefit across a range of clinically relevant probability thresholds. A two-sided *P* value < 0.05 was considered statistically significant for all analyses, except for *post hoc* tests, which used a Bonferroni-corrected threshold.

## Results

### Association between risk scores and AF incidence

**Figure 1:**
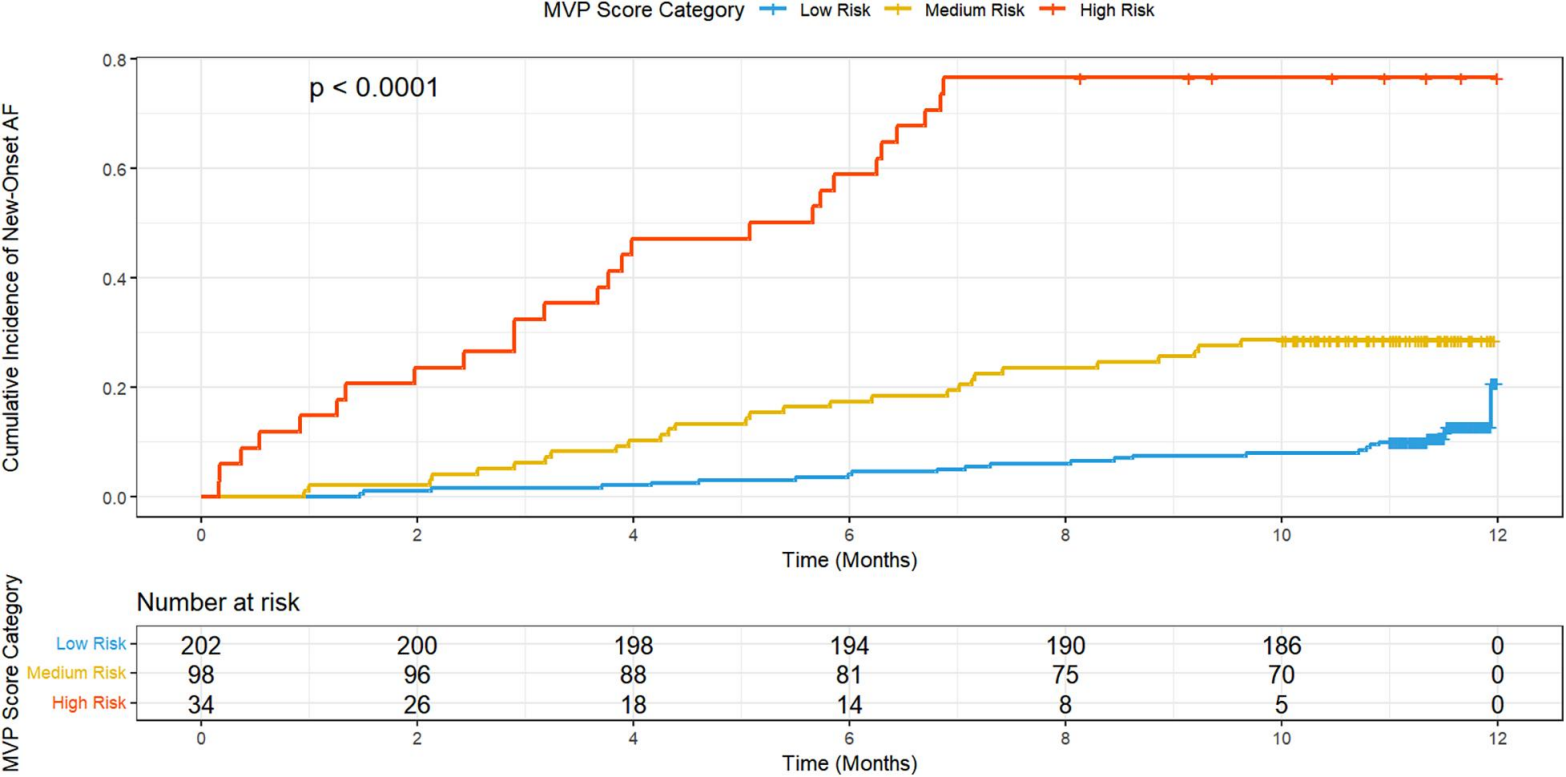
Kaplan–Meier cumulative incidence curves for new-onset atrial fibrillation (NOAF) stratified by MVP risk category. The curves illustrate the cumulative incidence of NOAF over the 12-month follow-up period for patients in the Low Risk (blue), Medium Risk (yellow), and High Risk (orange) categories. The High Risk group demonstrated a markedly elevated incidence rate (76.5%) with a steep early rise, consistent with high early-onset events. The differences between the three groups were statistically significant (Log-rank test *P* < 0.0001). The risk table below the *x*-axis displays the number of patients at risk at each time point. MVP, Morphology-Voltage-P wave duration.

In this study, 334 patients with AMI treated at our hospital from January 2018 to April 2021 were included. Patients were categorized into three groups based on their MVP scores derived from electrocardiograms: low-risk (202 cases), medium-risk (98 cases), and high-risk (34 cases). This classification was used to evaluate the applicability of the MVP score in predicting new-onset AF following AMI. During the 12-month follow-up period, the incidence of long-term AF was observed to be 11.9% in the low-risk group, 28.6% in the medium-risk group, and 76.5% in the high-risk group. Kaplan–Meier survival analysis visually confirmed these findings, illustrating a clear and significant separation in the cumulative incidence curves among the three groups (Log-rank *P* < 0.001, Figure 1). The high-risk group exhibited a rapid ascent in cumulative AF events compared to the gradual increase observed in the low- and medium-risk groups. These findings indicate a significant correlation between higher MVP scores and an increased risk of new-onset AF. The incidence of AF in the high-risk group was markedly higher, being several times greater than that in the low-risk group. This data underscores the predictive value of the MVP score, demonstrating its effectiveness in identifying patients at higher risk for AF post-AMI (Table 2).

**Table 2:** MVP ECG risk score for new-onset AF.

Risk category	Cases	MVP score	*P*-wave morphology	*P*-wave voltage	*P*-wave duration
Low risk	202	0–2	Normal	Normal	<120 ms
Medium risk	98	3–4	Abnormalities	Slightly elevated	120–200 ms
High risk	34	5–6 or above	Marked abnormalities	Significantly elevated	>200 ms

### Clinical and echocardiographic characteristics across MVP risk categories

A comprehensive analysis of various clinical features and echocardiographic parameters was performed to identify variables associated with the occurrence of AF among the study groups based on the MVP score. The median age significantly increased across risk categories, from 60 years in the low-risk group to 71 years in the high-risk group (*P* = 0.02). The proportion of male patients was similar across the groups. The prevalence of both hypertension (70.6% vs. 41.6% and 48.0%, *P* < 0.01) and diabetes mellitus (61.8% vs. 19.8% and 28.6%, *P* < 0.001) was significantly higher in the high-risk group compared to the low- and medium-risk groups. In the echocardiographic parameters, a significant trend was observed: the left atrial anteroposterior diameter increased from the low-risk group (40 mm) to the high-risk group (45 mm) (*P* < 0.001), and LVEF decreased across risk categories, with a median of 45% in the low-risk group, 40% in the medium-risk group, and 35% in the high-risk group (*P* < 0.001). The incidence of long-term AF was 11.9% in the low-risk group, 28.6% in the medium-risk group, and 76.5% in the high-risk group, demonstrating a significant increase in AF risk with higher MVP scores. The incidence of long-term AF was 11.9% in the low-risk group, 28.6% in the medium-risk group, and 76.5% in the high-risk group, demonstrating a significant increase in AF risk with higher MVP scores. When analyzed according to the timing of onset, early-onset NOAF (≤48 h) occurred in 4.0%, 10.2%, and 29.4% of patients in the low-, medium-, and high-risk groups, respectively. The incidence of late-onset NOAF (>48 h) was 7.9%, 18.4%, and 47.1% in the respective groups (all *P* for trend <0.001). The use of medications such as aspirin, beta-blockers, ACE inhibitors or ARBs, and statins did not differ significantly between the groups (Table 3).

**Table 3:** Baseline characteristics of the study population based on MVP risk scores.

Variables	Low risk (*n* = 202)	Medium risk (*n* = 98)	High risk (*n* = 34)	*P*
Age (years)	60 (54–66)	65 (58–72)	71 (65–78)	0.02
Gender				
Male	141 (69.8%)	64 (65.3%)	18 (52.9%)	0.48
Female	61 (30.2%)	34 (34.7%)	16 (47.1%)	0.37
Hypertension	84 (41.6%)	47 (48.0%)	24 (70.6%)	<0.01
Diabetes mellitus	40 (19.8%)	28 (28.6%)	21 (61.8%)	<0.001
Hyperlipidemia	108 (53.5%)	52 (53.1%)	15 (44.1%)	0.58
Smoking	88 (43.6%)	46 (46.9%)	13 (38.2%)	0.65
Chronic Renal Failure	10 (5.0%)	7 (7.1%)	3 (8.8%)	0.61
Coronary Artery Disease	140 (69.3%)	65 (66.3%)	23 (67.6%)	0.88
Risk scores				
CHA₂DS₂-VASc score	2 (1–3)	3 (2–4)	4 (3–5)	<0.001
C2HEST score	1 (0–2)	2 (1–3)	3 (2–4)	<0.001
ICD indication				
Primary	92 (45.5%)	24 (24.5%)	13 (38.2%)	<0.01
Secondary	162 (80.2%)	74 (75.5%)	21 (61.8%)	0.04
Device types				
WCD-ICD	102 (50.5%)	44 (44.9%)	13 (38.2%)	0.25
TV-ICD	60 (29.7%)	35 (35.7%)	13 (38.2%)	0.45
S-ICD	40 (19.8%)	19 (19.4%)	8 (23.5%)	0.83
Echocardiographic parameters				
LV ejection fraction (%)	45 (40–50)	40 (35–45)	35 (30–40)	<0.001
LV end-diastolic dimension(mm)	55 (50–60)	50 (45–55)	60 (55–65)	0.01
LV end-systolic dimension (mm)	35 (30–40)	33 (28–38)	40 (35–45)	<0.001
LA anteroposterior diameter (mm)	40 (35–45)	38 (33–43)	45 (40–50)	<0.001
New-onset atrial fibrillation				
Total NOAF, *n* (%)	24 (11.9%)	28 (28.6%)	26 (76.5%)	<0.001
Early-onset NOAF (≤48 h), *n* (%)	8 (4.0%)	10 (10.2%)	10 (29.4%)	<0.001
Late-onset NOAF (>48 h), *n* (%)	16 (7.9%)	18 (18.4%)	16 (47.1%)	<0.001
Out-hospital medication				
Aspirin	84 (41.6%)	35 (35.7%)	10 (29.4%)	0.25
Beta-blockers	56 (27.7%)	24 (24.5%)	8 (23.5%)	0.77
ACEI or ARB	34 (16.8%)	20 (20.4%)	8 (23.5%)	0.50
Statins	110 (54.5%)	44 (44.9%)	13 (38.2%)	0.07
Others	20 (9.9%)	15 (15.3%)	8 (23.5%)	0.05
Follow-up (months)	12 (10–13)	12 (9–13)	13 (10–14)	0.45

LV, left ventricular; LA, left atrial; ACEI, angiotensin-converting enzyme inhibitor; ARB, angiotensin II receptor blocker. MVP score criteria: *P*-wave morphology: 0 points (normal), 2 points (abnormal bifid/biphasic); *P*-wave voltage in lead I: 0 points (<0.1 mV), 1 point (0.1–0.15 mV), 2 points (>0.15 mV); *P*-wave duration in lead II: 0 points (<120 ms), 1 point (120–200 ms), 2 points (>200 ms).

### Electrocardiographic and laboratory correlates with MVP risk scores

In terms of electrocardiographic morphology, patients in the high-risk group predominantly exhibited bifid *P* waves (>120 ms), which was statistically significantly different from the other two groups, indicating a higher likelihood of experiencing AF risk in these patients. Additionally, the high-risk group showed a significant prolongation in *P*-wave duration [130 (120–140) ms], which was also statistically significant, further emphasizing the relevance of the MVP score in predicting AF risk. Among other laboratory variables, such as Urea and thyroid-stimulating hormone (TSH) levels, both showed significant increases in the high-risk group, potentially indicating increased atrial load and metabolic disturbances, which are also associated with an increased risk of AF (Table 4).

**Table 4:** Comparison of laboratory and ECG parameters across MVP score categories scores.

Variables	Low Risk	Medium Risk	High Risk	*P*
Hemoglobin (g/dL)	13.5 (12.9–14.1)	13.0 (12.4–13.6)	12.5 (12.0–13.0)	0.20
Lymphocytes (%)	3.0 (2.5–3.5)	2.8 (2.3–3.3)	2.5 (2.0–3.0)	0.15
WBC (cells/μL)	7.0 (6.5–7.5)	7.5 (7.0–8.0)	8.0 (7.5–8.5)	0.30
Platelet count (/mm^3^)	230 (210–250)	250 (230–270)	270 (250–290)	0.25
Creatinine (mg/dL)	0.9 (0.8–1.0)	1.1 (1.0–1.2)	1.3 (1.2–1.4)	0.05
Urea (mg/dL)	25 (20–30)	30 (25–35)	35 (30–40)	<0.001
TSH (mIU/L)	2.5 (2.0–3.0)	2.7 (2.3–3.1)	3.0 (2.5–3.5)	0.04
Albumin (mg/dL)	3.8 (3.5–4.1)	3.7 (3.4–4.0)	3.6 (3.3–3.9)	0.07
Glucose (mg/dL)	100 (95–105)	110 (105–115)	120 (115–125)	0.10
ECG morphology in inferior leads	Mostly non-biphasic	Mixed morphology	Mostly biphasic	<0.001
*P*-wave duration (ms)	110 (100–120)	120 (110–130)	130 (120–140)	<0.001

WBC, White Blood Cells.

### Comparison of predictive performance for new-onset atrial fibrillation

**Figure 2:**
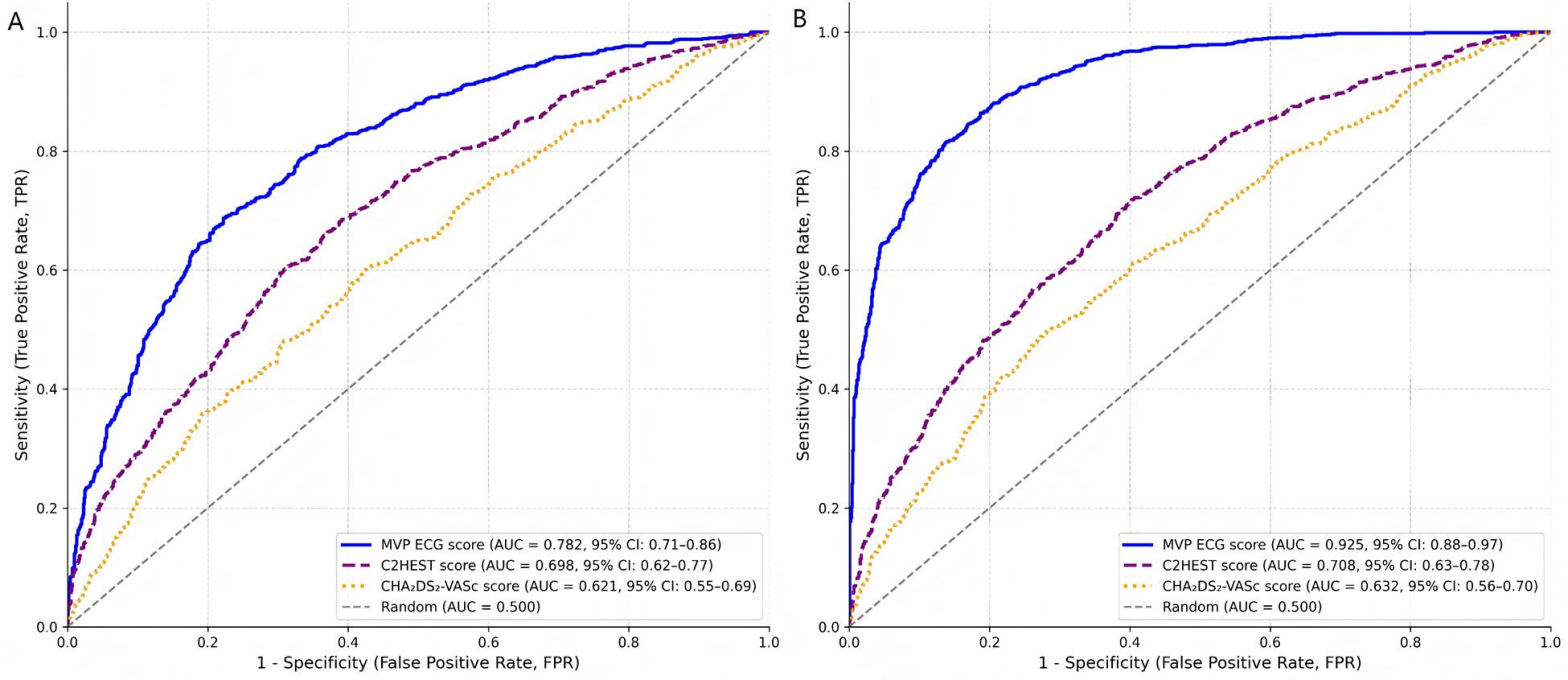
Receiver operating characteristic (ROC) curves for the prediction of early- and late-onset new-onset atrial fibrillation (NOAF). **(A)** ROC curves for early-onset NOAF (occurring ≤48 h after admission). **(B)** ROC curves for late-onset NOAF (occurring >48 h after admission). In both panels, the predictive performance of the MVP ECG score (solid blue line), the C2HEST score (dashed purple line), and the CHA₂DS₂-VASc score (dotted orange line) are shown. The corresponding areas under the curve (AUC) with 95% confidence intervals are: for early-onset NOAF—MVP: 0.782 (0.71–0.86), C2HEST: 0.698 (0.62–0.77), CHA₂DS₂-VASc: 0.621 (0.55–0.69); for late-onset NOAF—MVP: 0.925 (0.88–0.97), C2HEST: 0.708 (0.63–0.78), CHA₂DS₂-VASc: 0.632 (0.56–0.70). The diagonal dashed grey line represents the line of no discrimination (AUC = 0.5). The superior discriminative ability of the MVP score, particularly for late-onset NOAF, is visually evident.

The predictive performance of the MVP ECG risk score was systematically compared with both the CHA₂DS₂-VASc score and the C2HEST score for the prediction of new-onset atrial fibrillation. Performance was evaluated for total NOAF as well as stratified by timing of onset (early: ≤48 h; late: >48 h). For total NOAF, the MVP score demonstrated superior discriminative ability with an AUC of 0.908 (95% CI: 0.86–0.94), significantly outperforming both the CHA₂DS₂-VASc score (AUC 0.643, 95% CI: 0.58–0.72; *P* < 0.001) and the C2HEST score (AUC 0.715, 95% CI: 0.65–0.78; *P* < 0.001). When analyzing by onset timing, distinct patterns emerged. For early-onset NOAF, the MVP score maintained good predictive performance (AUC 0.782, 95% CI: 0.71–0.86) but showed less substantial advantage over the C2HEST score (AUC 0.698, 95% CI: 0.62–0.77; *P* = 0.04) (Table 5; Figure 2A). In contrast, for late-onset NOAF, the MVP score demonstrated exceptional discriminative ability (AUC 0.925, 95% CI: 0.88–0.97), significantly surpassing both the CHA₂DS₂-VASc score (AUC 0.632, 95% CI: 0.56–0.70; *P* < 0.001) and the C2HEST score (AUC 0.708, 95% CI: 0.63–0.78; *P* < 0.001) (Table 5; Figure 2B).

**Table 5:** Comparison of predictive performance of different risk scores for New-onset atrial fibrillation.

Prediction model	Outcome	AUC (95% CI)	*P*-value (vs. C2HEST)	*P*-value (vs. CHA₂DS₂-VASc)
MVP ECG Score	Total NOAF	0.908 (0.86–0.94)	<0.001	<0.001
	Early-onset NOAF (≤48 h)	0.782 (0.71–0.86)	0.04	<0.001
	Late-onset NOAF (>48 h)	0.925 (0.88–0.97)	<0.001	<0.001
C2HEST Score	Total NOAF	0.715 (0.65–0.78)	–	0.02
	Early-onset NOAF (≤48 h)	0.698 (0.62–0.77)	–	0.06
	Late-onset NOAF (>48 h)	0.708 (0.63–0.78)	–	0.04
CHA₂DS₂-VASc Score	Total NOAF	0.643 (0.58–0.72)	0.02	–
	Early-onset NOAF (≤48 h)	0.621 (0.55–0.69)	0.06	–
	Late-onset NOAF (>48 h)	0.632 (0.56–0.70)	0.04	–
Combined models		AUC (95% CI)	NRI (95% CI)	IDI (95% CI)
CHA₂DS₂-VASc + MVP	Total NOAF	0.914 (0.88–0.95)	0.35 (0.18–0.52)	0.08 (0.03–0.13)
C2HEST + MVP	Total NOAF	0.905 (0.87–0.94)	0.28 (0.12–0.44)	0.07 (0.02–0.12)

AUC, area under the receiver operating characteristic curve; NRI, net reclassification improvement; IDI, integrated discrimination improvement. *P*-values for AUC comparisons were derived from the DeLong test.

**Figure 3:**
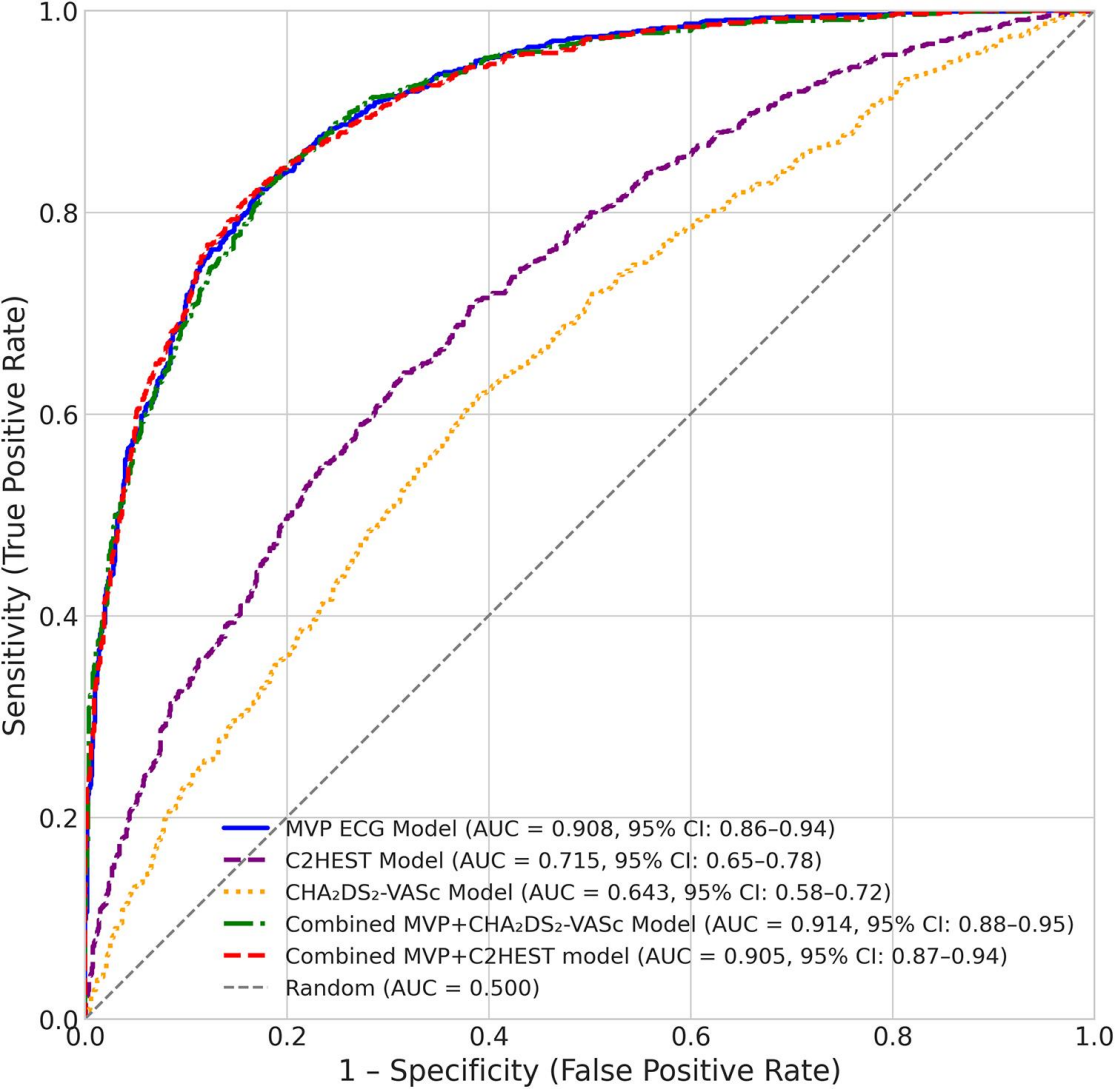
Receiver operating characteristic (ROC) curves comparing predictive performance of different models for total new-onset atrial fibrillation (NOAF). The ROC curves show the diagnostic performance of three single prediction models and two combined model: MVP ECG Model (solid blue line, AUC = 0.908, 95% CI: 0.86–0.94), C2HEST Model (dashed purple line, AUC = 0.715, 95% CI: 0.65–0.78), CHA₂DS₂-VASc Model (dotted orange line, AUC = 0.643, 95% CI: 0.58–0.72), the combined MVP + CHA₂DS₂-VASc Model (dash-dot green line, AUC = 0.914, 95% CI: 0.88–0.95), and the combined MVP + C2HEST model (AUC = 0.905, 95% CI: 0.87–0.94). The diagonal dashed grey line represents the line of no discrimination (AUC = 0.5). The *x*-axis represents 1–Specificity (False Positive Rate), and the *y*-axis represents Sensitivity (True Positive Rate).

**Figure 4:**
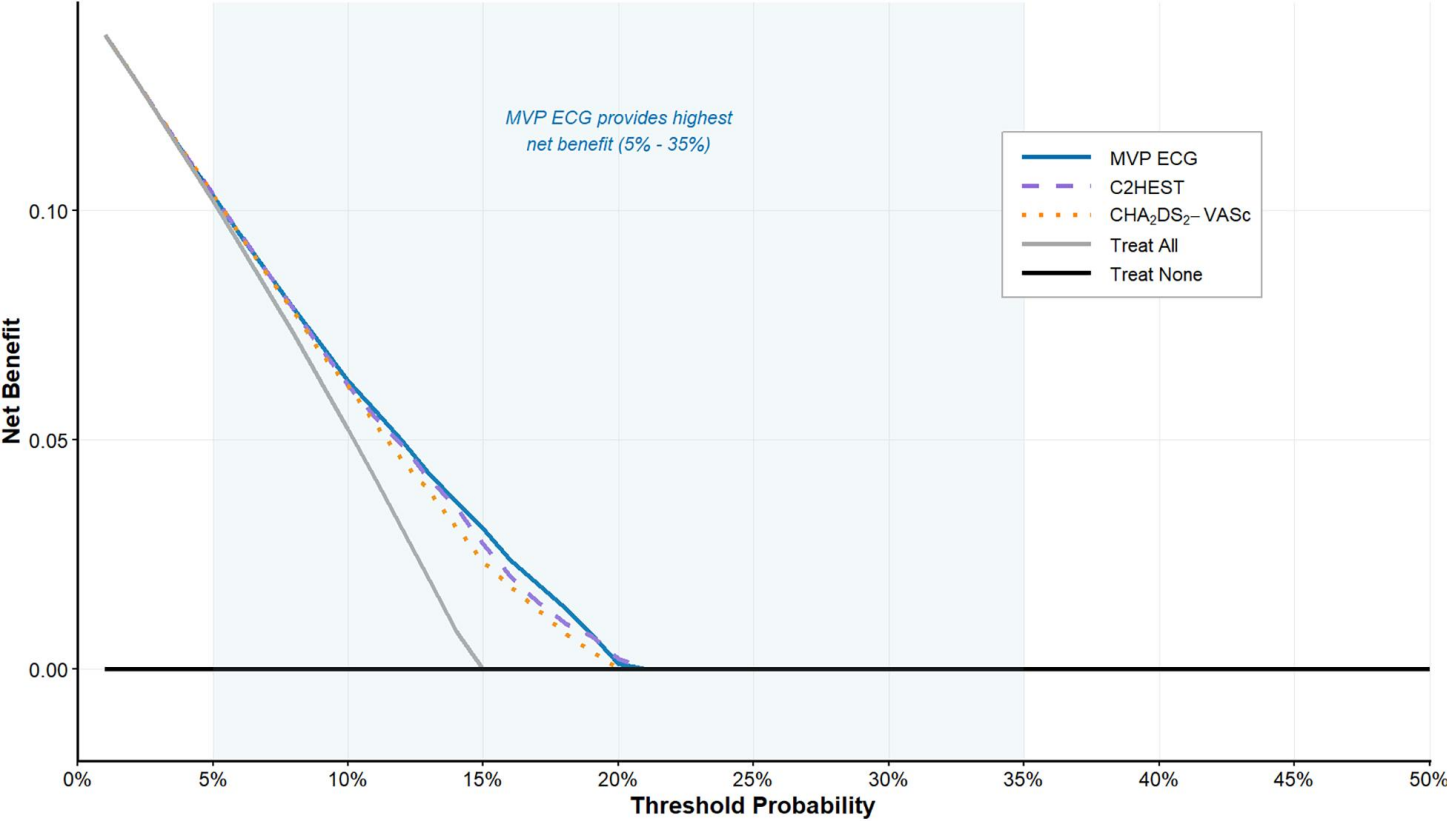
Decision curve analysis assessing the clinical utility of different models for predicting total new-onset atrial fibrillation. The figure displays the net clinical benefit derived from using different prediction models to inform decisions across a spectrum of threshold probabilities. Net benefit incorporates the relative clinical consequences of false-positive and true-positive predictions. The curves represent: the MVP ECG score (solid blue line), the C2HEST score (dashed purple line), the CHA₂DS₂-VASc score (dotted orange line), the strategy of intervening for all patients (“Treat All”; solid grey line), and the strategy of intervening for no patients (“Treat None”; horizontal black line). The MVP score provides the highest net benefit across threshold probabilities from approximately 5% to 35% (shaded area), demonstrating its superior clinical utility for risk-based decision-making compared to the clinical risk scores.

The addition of the MVP score to existing risk scores provided significant incremental predictive value. Combining the MVP score with the CHA₂DS₂-VASc score increased the AUC for total NOAF to 0.914 (*P* < 0.001), with a NRI of 0.35 (95% CI: 0.18–0.52, *P* < 0.001) and an IDI of 0.08 (95% CI: 0.03–0.13, *P* = 0.002). Similarly, adding the MVP score to the C2HEST score further improved discrimination for total NOAF (AUC increase from 0.715 to 0.905, *P* < 0.001), with an NRI of 0.28 (95% CI: 0.12–0.44, *P* < 0.001) and IDI of 0.07 (95% CI: 0.02–0.12, *P* = 0.003). These findings indicate that the MVP score captures unique electrophysiological information that complements both traditional comorbidity-based risk assessment and other AF-specific prediction tools (Table 5; Figure 3). To further evaluate the clinical utility of the MVP score beyond traditional discrimination metrics, we performed DCA for the prediction of total NOAF. DCA quantifies the net benefit of using a prediction model to guide clinical decisions across a range of probability thresholds, balancing the benefits of true-positive classifications against the harms of false positives. The results of decision curves for the MVP score, the C2HEST score, and the CHA₂DS₂-VASc score showed that across a wide and clinically relevant range of threshold probabilities (approximately 5% to 35%), using the MVP score for risk stratification provides a consistently higher net clinical benefit compared to the strategies of using either the C2HEST score, the CHA₂DS₂-VASc score, classifying all patients as positive (“Treat All”), or classifying none as positive (“Treat None”). The net benefit of the two clinical scores was modest and similar to each other, only marginally exceeding the “Treat All” strategy at higher thresholds (Figure 4). These results indicate that employing the MVP ECG risk score to guide decisions regarding intensified monitoring or preventive therapy in post-AMI patients would yield superior clinical outcomes compared to strategies based on existing clinical risk scores or universal approaches.

### Adjusted association of MVP risk categories with long-term AF incidence

The association between MVP risk categories and NOAF was further analyzed according to the timing of AF onset. The models were adjusted for age, gender, hypertension, diabetes mellitus, hyperlipidemia, smoking, chronic renal failure, coronary artery disease, CHA₂DS₂-VASc score, ICD indication, device types, echocardiographic parameters, and out-hospital medication.

In the multivariable Cox regression analysis for total NOAF, both the medium-risk (adjusted HR 2.2, 95% CI 1.2–3.9, *P* = 0.008) and high-risk (adjusted HR 5.8, 95% CI 3.2–10.5, *P* < 0.001) groups were associated with a significantly increased hazard compared to the low-risk group.

When stratifying by onset timing, similar patterns were observed but with notable differences in the magnitude of association. For early-onset NOAF (≤48 h), the medium-risk group showed an adjusted HR of 1.8 (95% CI 0.9–3.6, *P* = 0.08), while the high-risk group had an adjusted HR of 3.5 (95% CI 1.6–7.8, *P* = 0.002). For late-onset NOAF (>48 h), the medium-risk group demonstrated an adjusted HR of 2.5 (95% CI 1.3–4.8, *P* = 0.006), and the high-risk group showed the strongest association with an adjusted HR of 6.5 (95% CI 3.4–12.5, *P* < 0.001). These findings indicate that the MVP ECG risk score is not only a potent predictive tool for NOAF in AMI patients but also shows a particularly strong association with late-onset AF, which may reflect underlying atrial structural abnormalities. The score's predictive performance for both early and late-onset AF underscores its comprehensive clinical utility (Table 6).

**Table 6:** Cox regression models for total, early-onset, and late-onset NOAF based on MVP ECG risk scores.

Outcome	MVP risk category	Cases/Total	Case rate (%)	Unadjusted HR (95% CI)	Adjusted HR (95% CI)^a^
Total NOAF	Low Risk	24/202	11.9	1 (Reference)	1 (Reference)
	Medium Risk	28/98	28.6	2.4 (1.4–4.2)	2.2 (1.2–3.9)
	High Risk	26/34	76.5	6.4 (3.6–11.4)	5.8 (3.2–10.5)
Early-onset NOAF (≤48 h)	Low Risk	8/202	4.0	1 (Reference)	1 (Reference)
	Medium Risk	10/98	10.2	2.6 (1.3–5.4)	1.8 (0.9–3.6)
	High Risk	10/34	29.4	7.2 (3.3–15.7)	3.5 (1.6–7.8)
Late-onset NOAF (>48 h)	Low Risk	16/202	7.9	1 (Reference)	1 (Reference)
	Medium Risk	18/98	18.4	2.3 (1.2–4.4)	2.5 (1.3–4.8)
	High Risk	16/34	47.1	5.9 (3.1–11.2)	6.5 (3.4–12.5)

a Adjusted for age, gender, hypertension, diabetes mellitus, hyperlipidemia, smoking, chronic renal failure, coronary artery disease, CHA₂DS₂-VASc score, ICD indication, device types, echocardiographic parameters (LVEF, left atrial diameter), and out-hospital medication.

### Multivariate Cox regression analysis for predictors of long-term atrial fibrillation

**Figure 5:**
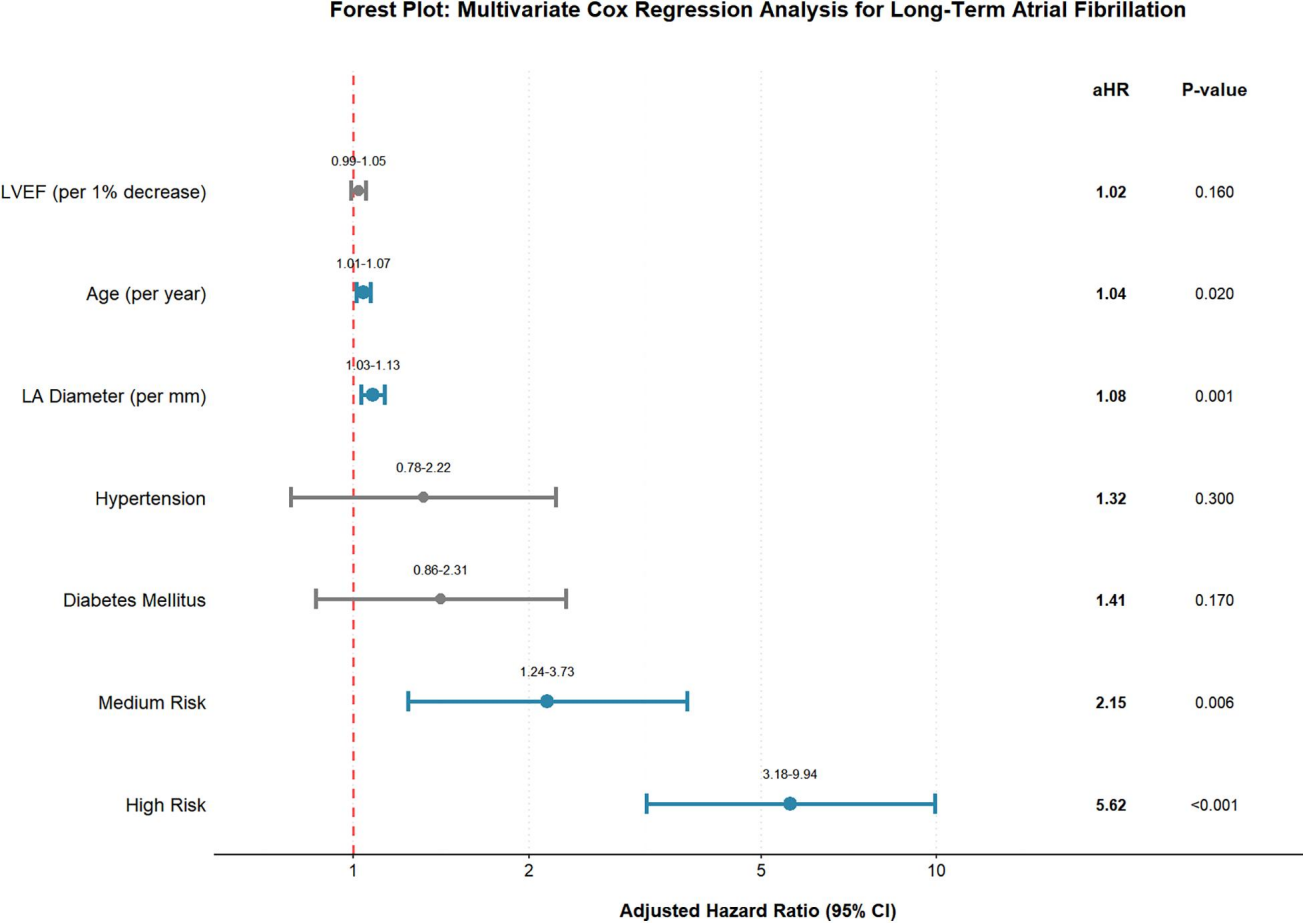
Forest plot of multivariate Cox regression analysis for predictors of long-term atrial fibrillation. The forest plot displays adjusted hazard ratios (aHR) and 95% confidence intervals from a multivariate Cox proportional hazards model. The model was adjusted for age, history of hypertension, history of diabetes mellitus, left atrial anteroposterior diameter, left ventricular ejection fraction, and MVP ECG risk category. Blue points and error bars indicate statistically significant predictors (*P* < 0.05), while gray indicates non-significant factors. The vertical dashed red line represents the reference line (HR = 1.0). Confidence intervals are displayed above the corresponding error bars. aHR, adjusted hazard ratio; LVEF, left ventricular ejection fraction; LA, left atrial; MVP, mitral valve prolapse.

To determine whether the MVP ECG risk score was an independent predictor of NOAF, we performed stratified multivariate Cox proportional hazards regression analyses. Variables with a *P*-value < 0.10 in the univariate analysis or those of established clinical relevance were included in the model. The final models were adjusted for age, history of hypertension, history of diabetes mellitus, left atrial anteroposterior diameter, LVEF, and MVP ECG risk category. After adjusting for these potential confounders, both a medium MVP risk score and a high MVP risk score remained strongly and independently associated with a significantly increased risk of NOAF. For total NOAF, the medium-risk group had an adjusted Hazard Ratio (aHR) of 2.15 (95% CI 1.24–3.73, *P* = 0.006) and the high-risk group had an aHR of 5.62 (95% CI 3.18–9.94, *P* < 0.001). When analyzing by onset timing, the MVP score showed differential predictive strength. For early-onset NOAF, the high-risk group maintained significant association (aHR 3.42, 95% CI 1.52–7.70, *P* = 0.003), while for late-onset NOAF, both medium-risk (aHR 2.34, 95% CI 1.29–4.24, *P* = 0.005) and high-risk (aHR 6.28, 95% CI 3.31–11.93, *P* < 0.001) groups showed strong independent associations. Among the covariates, increased age (aHR 1.04 per year, 95% CI 1.01–1.07, *P* = 0.02) and enlarged left atrial diameter (aHR 1.08 per mm, 95% CI 1.03–1.13, *P* = 0.001) were also identified as significant independent predictors of AF (Figure 5). The stronger association between MVP score and late-onset AF suggests that the MVP score captures atrial substrate abnormalities that are particularly relevant to the development of sustained AF beyond the acute phase of AMI.

## Discussion

This study demonstrates the significant prognostic value of the MVP ECG risk score for predicting NOAF in 334 patients with acute myocardial infarction. We observed a graded increase in AF incidence across MVP risk categories, with the high-risk group exhibiting markedly elevated risk. The score's strong predictive performance persisted after comprehensive adjustment for clinical and echocardiographic confounders, underscoring its utility as an independent risk stratification tool. Atrial fibrillation substantially increases morbidity and mortality following myocardial infarction (25), highlighting the clinical importance of early identification of high-risk patients. The MVP score provides a simple, non-invasive method for identifying these patients in routine practice, facilitating targeted monitoring and potential preventive interventions. Our findings align with existing literature documenting the association between *P*-wave abnormalities and AF risk (26), while extending this evidence to the post-AMI population where timely risk assessment is particularly crucial.

Our findings robustly demonstrate that the MVP ECG risk score serves as a strong and independent predictor of new-onset AF in patients with AMI, even after comprehensive adjustment for established clinical, echocardiographic, and laboratory confounders. The graded increase in AF risk across MVP categories, with the high-risk group exhibiting a markedly elevated hazard, underscores the score's ability to quantify the electrophysiological and structural atrial substrate predisposing to AF post-AMI. This aligns with existing literature emphasizing the prognostic value of *P*-wave abnormalities for AF risk stratification (27). The MVP score integrates key atrial electrical parameters (morphology, voltage, duration), which may reflect underlying atrial fibrosis, enlargement, and conduction heterogeneity—pathophysiological hallmarks promoting AF (28). Importantly, our stratified analysis revealed that the predictive strength of the MVP score was more pronounced for late-onset NOAF (occurring >48 h post-AMI) compared to early-onset events. This differential performance supports the pathophysiological premise that the MVP score primarily captures chronic alterations in atrial substrate such as fibrosis and electrical remodeling, which are central to the development of sustained AF beyond the immediate ischemic insult (8). Our study further underscores the distinct nature of early and late NOAF, which are associated with different prognostic implications and underlying mechanisms. Prior research indicates that early NOAF is frequently linked to acute atrial ischemia, such as occlusion of atrial branches, whereas late NOAF is more related to factors like left ventricular dysfunction and systemic inflammation, and is associated with a higher risk of adverse in-hospital events and mortality (24, 29). Furthermore, the persistence of MVP's predictive power after adjusting for factors like left atrial diameter and inflammation (30) suggests it provides unique information beyond traditional risk markers. Its simplicity and derivation from a standard ECG make it a practical tool for bedside risk assessment, potentially identifying AMI patients who benefit from intensified monitoring and early preventive strategies to mitigate AF-related complications.

The CHA₂DS₂-VASc score is a well-validated clinical tool originally developed for stroke risk stratification in patients with established atrial fibrillation, assessing thromboembolic risk based on clinical comorbidities including Congestive heart failure, Hypertension, Age ≥75 years, Diabetes mellitus, Stroke history, Vascular disease, Age 65–74 years, and Sex category (31). While it has been subsequently applied to predict new-onset AF in various clinical contexts, its performance remains suboptimal in specific populations such as post-AMI patients. Similarly, the C2HEST score, a tool specifically designed for AF risk prediction, also demonstrated only modest discriminative ability in our cohort. Our analysis demonstrates that the MVP ECG risk score not only predicts new-onset AF effectively but also provides significant incremental value beyond these established clinical scores. The superior discriminative ability, particularly for late-onset AF, and the significantly improved net reclassification observed when combining the MVP score with either clinical tool suggest that it captures distinct pathophysiological elements of AF risk. Specifically, the MVP score appears to quantify direct, ECG-visible atrial electrical and structural abnormalities that are not fully reflected in scores based solely on clinical comorbidities or historical data (32). This electrophysiological profiling may be particularly relevant in the AMI population, where acute ischemic injury can exacerbate pre-existing atrial substrate vulnerability (33). The findings align with growing evidence that direct electrocardiographic markers of atrial cardiopathy offer complementary risk stratification value to traditional clinical predictors. By enabling more precise identification of high-risk patients, the MVP score could help prioritize individuals who may benefit from intensified monitoring or preemptive anticoagulation strategies, potentially improving clinical outcomes in this vulnerable population (17, 25). Future studies should validate these findings in larger cohorts and explore the utility of MVP-guided management protocols.

The DCA further supports the clinical utility of the MVP score, demonstrating that its use for risk stratification provides greater net benefit than management strategies based on existing clinical scores alone across realistic threshold probabilities. Beyond its predictive accuracy, the clinical value of the MVP score lies in its potential to guide personalized management strategies. Although our study does not evaluate clinical outcomes of interventions, a plausible pathway can be hypothesized. For instance, high-risk patients identified by the MVP score could be prioritized for more intensive and prolonged rhythm monitoring, earlier initiation of oral anticoagulation based on a higher CHA₂DS₂-VASc score and the presence of atrial cardiopathy, or more aggressive upstream therapy targeting atrial remodeling (e.g., with renin-angiotensin-aldosterone system inhibitors). The ability of the MVP score to further stratify risk beyond clinical scores like C2HEST could refine such targeted approaches. Such a risk-based approach has been shown to improve the efficiency of screening and management in other cardiovascular contexts (34, 35). Future randomized controlled trials are essential to investigate whether implementing a MVP score-based algorithm, which triggers specific monitoring and preventive interventions, can ultimately reduce the incidence of AF-related complications, such as stroke and heart failure hospitalization, in patients post-AMI.

However, our study has certain limitations. Firstly, the high discriminative performance of the MVP score (AUC 0.908) observed in our cohort, while encouraging, should be interpreted with caution. Although our model was internally validated using standard statistical methods and remained robust after multivariable adjustment, the elevated AUC may reflect a degree of overfitting inherent to single-center, retrospective studies with moderate sample sizes. The MVP score incorporates atrial electrical parameters that are mechanistically linked to AF substrate, which may explain its strong predictive ability in this homogeneous AMI population. However, such performance may not fully generalize to broader, more heterogeneous populations treated in different settings. Overfitting is a recognized limitation of predictive models developed and tested within the same cohort, particularly in the absence of external validation. Similar high AUC values have been reported in other single-center ECG-based AF prediction studies, underscoring the need for external validation before clinical implementation. The retrospective and observational design may introduce selection bias and unmeasured confounding. Although we performed rigorous statistical adjustments, residual confounding cannot be excluded. Therefore, our findings should be viewed as hypothesis-generating rather than conclusive. Future large-scale, multi-center prospective studies are essential to externally validate the MVP score's performance, calibrate its risk thresholds, and assess its generalizability across diverse patient populations and healthcare systems. Furthermore, our method for detecting long-term AF relied on scheduled clinic visits with ECGs and healthcare system records, rather than continuous cardiac monitoring (e.g., with implantable loop recorders). While this reflects real-world clinical practice and captures AF episodes that are typically clinically significant, it may have resulted in under-detection of asymptomatic or brief paroxysmal AF. This potential ascertainment bias could lead to an underestimation of the true incidence of AF, particularly in lower-risk groups, and might attenuate the observed strength of association between the MVP score and AF risk. Nevertheless, the AF events identified in this study are precisely those that would trigger clinical evaluation and management in routine practice, enhancing the practical relevance of our findings.

Secondly, excluding patients with implanted cardiac devices was necessary in order to ensure that the *P*-wave could be analysed correctly using surface ECG. However, this exclusion limits the applicability of our findings to a clinically important and high-risk subgroup of AMI patients. Patients requiring device therapy, such as those with severe conduction disease or cardiogenic shock, represent a population at potentially elevated risk for both AMI complications and atrial fibrillation. Our results, therefore, may not be generalizable to this more complex patient spectrum. Future prospective studies designed to validate the MVP score should endeavor to include these patients, potentially utilizing device-stored electrograms to adjudicate AF episodes, to determine the tool's utility across the full AMI population. Finally, While the MVP score demonstrates strong predictive performance, its current reliance on manual *P*-wave measurement raises important practical considerations for clinical translation. As noted in our methods, we observed good inter-observer agreement through rigorous training and adjudication processes (ICC > 0.89 for continuous measures, *κ* = 0.85 for morphology). Nevertheless, in routine clinical practice, manual measurement can be time-consuming and may be subject to greater variability, potentially affecting reproducibility and scalability. To facilitate widespread adoption, the future development and integration of automated or semi-automated algorithms for MVP score calculation are crucial. Recent advances in computerized ECG analysis and artificial intelligence have shown promise in accurately quantifying complex waveform features, including *P*-wave parameters (36). Embedding the MVP algorithm within modern ECG management systems could provide real-time, standardized risk assessment at the point of care, minimizing observer-dependent variability and workflow disruption. Before such integration, further studies are needed to validate the performance of automated MVP measurements against the manual gold standard and to confirm that the automated score retains its predictive validity. The ultimate goal is to transform the MVP score from a research tool into a seamless, efficient component of routine post-AMI evaluation. In light of these limitations, our findings should be interpreted as generating a clinically promising hypothesis rather than providing definitive evidence. We strongly emphasize the need for rigorous external validation in prospective, multi-center studies that include diverse patient populations, encompassing those with and without cardiac devices, to confirm the predictive performance and calibrate the risk thresholds of the MVP score for widespread clinical use.

## Conclusion

In conclusion, the MVP ECG score is a robust and independent predictor of NOAF in patients with acute myocardial infarction. Our findings demonstrate its superior discriminative ability compared to both the CHA₂DS₂-VASc and C2HEST scores, with particular strength in predicting late-onset AF, and significant incremental prognostic value when combined with established clinical risk factors. This simple, non-invasive tool enables early identification of high-risk patients who may benefit from intensified monitoring and preventive strategies, potentially improving clinical outcomes in this vulnerable population. Future randomized studies are warranted to evaluate the efficacy of MVP score-guided management protocols in reducing AF-related complications.

## Data Availability

The original contributions presented in the study are included in the article/Supplementary Material, further inquiries can be directed to the corresponding author.
